# Volatile Organic Compounds of *Streptomyces* sp. TOR3209 Stimulated Tobacco Growth by Up-Regulating the Expression of Genes Related to Plant Growth and Development

**DOI:** 10.3389/fmicb.2022.891245

**Published:** 2022-05-20

**Authors:** Yuxi He, Wenyu Guo, Jieli Peng, Jinying Guo, Jia Ma, Xu Wang, Cuimian Zhang, Nan Jia, Entao Wang, Dong Hu, Zhanwu Wang

**Affiliations:** ^1^Institute of Agro-Resources and Environment/Hebei Fertilizer Technology Innovation Center, Hebei Academy of Agriculture and Forestry Sciences, Shijiazhuang, China; ^2^School of Landscape and Ecological Engineering, Hebei University of Engineering, Handan, China; ^3^Departamento de Microbiología, Escuela Nacional de Ciencias Biológicas, Instituto Politécnico Nacional, Mexico City, Mexico

**Keywords:** RNA sequencing, SPME, *Streptomyces*, plant growth-promoting rhizobacteria (PGPR), volatile organic compounds (VOCs)

## Abstract

To investigate the mechanism underlying the plant growth-promoting (PGP) effects of strain *Streptomyces* sp. TOR3209, PGP traits responsible for indoleacetic acid production, siderophore production, and phosphate solubilization were tested by culturing the strain TOR3209 in the corresponding media. The effects of volatile organic compounds (VOCs) produced by the strain TOR3209 on plant growth were observed by co-culturing this strain with tobacco seedlings in I-plates. Meanwhile, the effects of VOCs on tobacco gene expression were estimated by performing a transcriptome analysis, and VOCs were identified by the solid-phase micro-extraction (SPME) method. The results showed positive reactions for the three tested PGP traits in the culture of strain TOR3209, while the tobacco seedlings co-cultured with strain TOR3209 revealed an increase in the fresh weight by up to 100% when compared to that of the control plants, demonstrating that the production VOCs was also a PGP trait. In transcriptome analysis, plants co-cultured with strain TOR3209 presented the highest up-regulated expression of the genes involved in plant growth and development processes, implying that the bacterial VOCs played a role as a regulator of plant gene expression. Among the VOCs produced by the strain TOR3209, two antifungal molecules, 2,4-bis(1,1-dimethylethyl)-phenol and hexanedioic acid dibutyl ester, were found as the main compounds. Conclusively, up-regulation in the expression of growth- and development-related genes via VOCs production is an important PGP mechanism in strain TOR3209. Further efforts to explore the effective VOCs and investigate the effects of the two main VOCs in the future are recommended.

## Introduction

In the context of population growth and rapid economic development, chemical fertilizers/pesticides have been used in agriculture to increase production, while the excessive use of these chemicals has caused serious negative impacts on the agricultural ecosystems (Vejan et al., 2016). To reduce the application of chemical fertilizers/pesticides while maintaining high production, environment-friendly biofertilizers and biopesticides have been explored (Parewa et al., 2021). In the development of biofertilizers and biopesticides, microorganisms and their metabolites have received a lot of attention, since their interactions with the plants could improve the growth and production (Saharan and Nehra, 2011; Bhattacharyya and Jha, 2012) by various mechanisms, as mentioned in the following sections. In general, all the microbes with plant growth-promoting (PGP) abilities are called PGP microorganisms; among them, the bacteria colonized in the rhizosphere, so-called PGP rhizobacteria (PGPR), have been studied extensively and applied widely (Basu et al., 2021; Mohanty et al., 2021).

Many studies on PGPR have been focused on the capacities of the bacteria to promote plant health, such as growth-enhancing potential, stress tolerance, and the production of useful biologically active secondary metabolites (Spaepen and Vanderleyden, 2011). PGPR can stimulate plant growth by various mechanisms, such as nitrogen fixation (Nassar et al., 2005), phosphate dissolution, siderophore production, antibiotic secretion, and production of plant beneficial volatile organic compounds (VOCs) (Oleńska et al., 2020). They can also promote nutrient availability, nutrient uptake (Pailan et al., 2015), and produce plant hormones, such as auxins (Lee et al., 2012), cytokinins (Lugtenberg and Kamilova, 2009), abscisic acid (Sgroy et al., 2009), and gibberellins (Khan et al., 2014). These abilities can significantly help and improve the growth of their host plants. Therefore, the PGP microorganisms, such as *Rhizobium, Bacillus, Pseudomonas, Streptomyces*, and *Trichoderma*, and their metabolites have attracted increasing attention in the production of biological fertilizers and/or biopesticides (Atieno et al., 2020).

The members of the genus *Streptomyces* are Gram-positive, spore-forming bacteria that are widely distributed in the soil and other habitats, and they are well known for their ability to produce a large number of bioactive secondary metabolites (antibiotics) (Quinn et al., 2020), but rarely act as phytopathogens (Li et al., 2019). Recently, *Streptomyces* strains have been utilized as PGPR for several crops, including wheat (Akbari et al., 2020), tomato (Hu et al., 2020), chickpea (Gopalakrishnan et al., 2015), sorghum (Gopalakrishnan et al., 2013), and rice (Suárez-Moreno et al., 2019). The PGP effects of these *Streptomyces* strains have been attributed to their production of siderophores and indole-3-acetic acid, nitrogen fixation, and phosphate solubilization, which make them potential biofertilizers (Romano-Armada et al., 2020). In addition, some of the *Streptomyces* strains could improve plant growth and production by increasing the plant resistance to phytopathogens and to the abiotic stresses, which is related to their production of chitinolytic enzymes (inhibiting insects and fungi), induction of non-specific plant resistance, production of bioactive compounds (VOCs), and antibiotic/siderophore production (Romano-Armada et al., 2020). From *Streptomyces* species, more than 120 volatile substances belonging to alkenes, esters, alcohols, and ketones have been detected (Schöller et al., 2002), and various functions of these VOCs have been reported in agriculture, including PGP effects and phytopathogen control (Olanrewaju and Babalola, 2019). However, the mechanism by which the VOCs produced by *Streptomyces* promote plant growth and development is unclear.

Volatile organic compounds are secondary metabolites produced by many fungi and bacteria (Korpi et al., 2009), which are believed to act as signal molecules in interspecific and intraspecific interactions and in intercellular communication (Schmidt et al., 2015). Therefore, the in-depth study on the effects of VOCs on plant gene expression might provide some insight into the interactions between microbes and plants, as well as into the exploration of biofertilizers/biopesticides. Previously, *Streptomyces* sp. TOR3209 was isolated from the rhizosphere of tomato as a PGP bacterium (Hu et al., 2012), and the inoculation of this strain could significantly promote the growth and enhance the yield of tomato and other crops by changing the structure of the microbial community in the rhizosphere (Hu et al., 2020). However, its capacity to produce VOCs and whether the VOCs play a role in inducing the PGP effects have not been revealed. To check if the VOC production is one of the mechanisms of PGP effects in the strain TOR3209 and to estimate the effects of plant metabolism, we evaluated the PGP ability of VOCs produced by *Streptomyces* sp. TOR3209 and investigated the molecular composition of these VOCs. We also identify the important molecular events involved in the rhizosphere bacteria–plant interaction by transcriptome analysis.

## Materials and Methods

### Incubation of Bacteria and Inoculant Preparation

The strain *Streptomyces* sp. TOR3209 originally isolated from the rhizosphere of tomato plants (Hu et al., 2012) was used in this study, considering its role in the promotion of growth in tomatoes and other plants (Hu et al., 2020). To prepare the inoculant, the strain TOR3209 was massively inoculated on plates containing the Gause's No.1 medium (soluble starch 20 g, KNO_3_ 1.0 g, K_2_PO_4_ 0.5 g, MgSO_4_·7H_2_O 0.5 g, NaCl 0.5 g, FeSO_4_·7H_2_O 0.01 g, Agar 20 g, in 1 L of distilled water, pH 7.2–7.4) and incubated at 30°C for 24 h. Then the bacterial biomass was scraped off the plate, suspended in sterilized 10 mM MgCl_2_, and adjusted to 10^8^–10^9^ CFU/ml based on both optical density (OD_600_ = 1.0) and plate count. To test the effects of VOCs, strain TOR3209 was cultured overnight in Gause's No.1 liquid medium at 30°C with shaking at 180 rpm.

### Plant Growth-Promoting Test

To estimate the PGP effects of strain TOR3209 or its VOCs, tobacco (*Nicotiana benthamiana* Domin) was used as a model plant, which showed a positive response to inoculation of strain TOR3209 in our previous study. The seeds of tobacco were purchased from Huayueyang Biotechnology (Beijing) Co., Ltd.

#### Growth Promotion of Plants by Inoculation

The seeds were surface sterilized with 95% ethanol for 30 s, 2% (v/v) sodium hypochlorite for 5 min, and then washed with sterile distilled water (SDW) three times as described previously (Hu et al., 2020). The surface-sterilized seeds were germinated on 0.5 × Murashige and Skoog (MS) medium (PhytoTechnology, United States) supplemented with 1.5% (w/v) sucrose and 1.8% agar (w/v). The MS agar plates with seeds were kept at 4°C for 12 h for vernalization and then kept in a plant growth chamber for germination at 25°C under a long-day photoperiod (day/night: 16/8 h) with a light intensity of 100 μmol m^−2^ s^−1^. After 5 days of incubation, the seedlings were transferred to a 24-well plate containing the MS agar and incubated in the plant growth chamber with 16 h light/8 h dark conditions. Five days later, tobacco plants in the plate were inoculated with 20 μl of inoculant (10^8−9^ CFU/ml) around the root, and the negative control plants were inoculated with 20 μl of sterilized 10 mM MgCl_2_ solution. The tobacco seedlings were harvested 5 days after the inoculation, and total fresh weight was measured. The experiment was repeated three times, and 10 replications (seedlings) for each treatment were included each time.

#### Growth Promotion of Plants by VOCs of Strain TOR3209

Previously, the VOCs of bacteria have been reported as novel agents in promoting plant growth (Audrain et al., 2015). To evaluate the growth-promoting effect of VOCs on the tobacco plants, specialized plastic Petri dishes (I-plates with 9 cm in diameter) were used, which were divided by a partition into two equal chambers. In these I-plates, *Streptomyces* sp. TOR3209 and tobacco plants could be co-cultivated without direct contact. One chamber of the I-plate contained half-strength MS agar, and the other chamber contained Gause's No.1 medium with a sterile filter paper disk (8 mm in diameter) on its surface. After 5 days of germination as mentioned earlier, five seedlings were transferred to the chamber containing MS agar. After 3 days of plant growth, 20 μl of overnight incubated strain TOR3209 (see section Incubation of Bacteria and Inoculant Preparation) or sterilized distilled water (SDW) for treatment and control (CK), respectively, were applied drop-wise onto the filter paper disk in the chamber containing Gause's No.1 medium. Then, the I-plates were sealed with parafilm and cultured in the growth chamber under the day/night cycle of 16/8 h, with a light intensity of 100 μmol m^−2^ s^−1^ at 25°C. The fresh weight of each plant was measured after 1 week of growth. The experiment was repeated independently for three times, and 10 replications (seedlings) per treatment were included each time.

### Characterization of Plant Growth-Promoting Traits

To estimate the possible PGP mechanism of strain TOR3209, the PGP traits mentioned by Romano-Armada et al. (2020), such as the formation of indole-3-acetic acid (IAA), production of siderophores, and solubilization of phosphate, were measured for this strain.

#### Auxin Production Assay

The production of IAA was quantified by the colorimetric method using the Salkowski reagent (50 ml of 35% HClO_4_ and 1 ml of 0.5 M FeCl_3_) (Glickmann and Dessaux, 1995). The strain TOR3209 was activated in LB (Difco) broth for 24 h, and then a full loop of the culture was inoculated in 5 ml of the LB broth for culturing at 30°C with shaking at 180 rpm for 16 h. Then, 20 μl of bacterial suspension was transferred to 5 ml of LB broth supplemented with 0.1% L-tryptophan. After 16 h of incubation, 1 ml of the bacterial culture was centrifuged at 8,000 rpm for 10 min to remove the cells, and 300 μl of the supernatant was mixed with 600 μl of Salkowski reagent. The mixture was left in the dark for 30 min to develop the color (Phi et al., 2010). After the reaction, the absorbance of the mixture was measured at 540 nm using a UV–visible spectrophotometer (MAPADA, China). The concentration of IAA in the culture was calculated (Sigma, USA) based on the standard curve of IAA (serial dilutions from 0 to 50 μg/ml). The experiment was repeated three times, and 10 replications were included for each treatment each time.

#### Siderophore Production

In this analysis, siderophore production was checked as described previously (Neilands, 1987). Briefly, the strain TOR3209 was cultured on Chrome Azurol S (CAS) agar (Pérez-Miranda et al., 2007) and incubated at 30°C for 7 days. The formation of a yellow ring around the colony indicates the production of siderophore. To determine the siderophore type, a FeCl_3_ test was performed (Chowdappa et al., 2020). Briefly, the strain was cultured in 5 ml of CAS broth at 30°C with rotation for 5 days, and 1 ml of supernatant of the culture obtained by centrifugation (12,000 rpm for 1 min) was mixed with 2 ml of FeCl_3_ solution (2%, w/v). Then the absorption was scanned from 300 to 600 nm with a spectrophotometer (MAPADA, China). The occurrence of a peak between 420 and 450 nm and a peak at 495 nm demonstrate the presence of hydroxamate and catecholate types of siderophores, respectively. The CAS broth without inoculation was used as a blank to detect the siderophore type. The experiment was repeated three times, and 10 replications were included for each treatment each time.

#### Phosphate Solubilization

A single colony of strain TOR3209 grown on Gause's No. 1 medium at 30°C for 3 days was inoculated in an organic phosphorus medium (Jiwen et al., 2011) and an inorganic phosphorus (PKO) medium (Nautiyal, 1999), respectively, to measure the phosphorus solubilization. The inoculated plates were cultured at 30°C for 12 days, and the formation of a transparent ring surrounding the colony demonstrated the dissolution of phosphorus. Since the organophosphorus component in the medium is prepared using egg yolk (lecithin), it may undergo denaturation during long-term culture. Therefore, a control plate was set up for the organophosphorus solubilization to avoid false-positive results. The experiment was repeated three times, and 10 replications were included for each treatment each time.

### Expression of Plant Genes Responding to the VOCs

After 7 days of exposure to VOCs (co-cultured with strain TOR3209 in I-plates), as mentioned in Section Growth Promotion of Plants by VOCs of Strain TOR3209, the aerial parts (leaves and stems) of tobacco seedlings were collected from three I-plates of treatment or control in triplicate (15 plants in total for treatment or control). Experiments for treatments and controls were repeated three times per group. Total RNA was extracted from the plant samples using the TIANGEN Magnetic Tissue/Cell/Blood Total RNA Kit (DP762- T1A), according to the manufacturer's guidelines. The concentration of RNA in the extracts was determined using a NanoDrop spectrophotometer (Thermo Scientific), and the quality and integrity of RNA in the extracts were tested by agarose gel electrophoresis (Laila et al., 2017). For each extract, 3 μg of RNA was used as input material to construct the sequencing library using the NEBNext Ultra II RNA Library Prep Kit for Illumina (#E7775). The procedure described by Laila et al. (2017) was followed. Briefly, mRNAs were purified from total RNAs using poly-T oligo-attached magnetic beads. Fragmentation was carried out using divalent cations under elevated temperature in an Illumina proprietary fragmentation buffer. The first strand of cDNA was synthesized using random oligonucleotides and SuperScript II. Synthesis of the second strand of cDNA was subsequently performed using DNA polymerase I and RNase H. Remaining overhangs were converted into blunt ends via exonuclease/polymerase activities, and the enzymes were later removed. After adenylation of the 3′ ends of the DNA fragments, Illumina PE adapter oligonucleotides were ligated to prepare for hybridization. To select the cDNA fragments of the preferred size (200 bp), the library fragments were purified using the AMPure XP system (Beckman Coulter, Beverly, CA, USA). DNA fragments with ligated adaptor molecules on both ends were selectively enriched using Illumina PCR Primer Cocktail in a 15-cycle PCR reaction. Products were purified (AMPure XP system) and quantified by performing the Agilent High Sensitivity DNA assay on a Bioanalyzer 2100 system (Agilent). The sequencing library was then sequenced commercially on a Novaseq platform (Illumina) by Shanghai Personal Biotechnology Cp. Ltd. Filtration of high-quality reads by eliminating low-quality reads (Q <20), removal of adapter, assembling of the transcriptome, and quantification of transcript abundances were performed as described by Howlader et al. (2020).

The gene expression levels between the samples subjected to VOC treatment and the control were estimated by using fragments per kilobase of transcript per million fragments mapped (FPKM) values, as described by Howlader et al. (2020). In the transcription group, we generally took into account FPKM > 1 when the gene was expressed. Pearson correlation coefficients were used to determine the level of correlation between the expression of genes in the samples to test the reliability of the experiments and whether sample selection was reasonable (van Ruissen et al., 2005). The log2 fold change was calculated by dividing the FPKM values of the treatment groups by the values of the control group. High-quality reads were processed using the Perl script, and the differentially expressed genes (DEGs) were identified using the edgeR package (Boscari et al., 2013), taking a fold change ≥ 2 as the threshold value.

The cluster analysis of FPKM-normalized expression values was carried out, as mentioned by Howlader et al. (2020), to differentiate the up- and down-regulated transcripts between the VOC treatment and the control samples, by using the cluster software version 3.0 and the Java TreeView to visualize the dendrogram. For GSEA, the KEGG pathway collection (Homo sapiens (human) Release 53.0) (Kanehisa and Goto, 2000) was used to define the gene sets using the GSEABase package in Bioconductor/R version 2.9.2 (Gentleman, 2004). Gene ontology (GO) and the Kyoto Encyclopedia of Genes and Genomes (KEGG) pathway enrichment analysis were used to characterize the differentially expressed genes, and GO functional annotations were obtained from the non-redundant annotation results using the Blast2GO software (Conesa et al., 2005).

### Extraction and Identification of VOCs

The strain TOR3209 was activated by culturing in Luria-Bertani (LB) broth (NaCl 10 g, tryptone 10 g, yeast extract 5 g, distilled water 1 L, pH 6.8–7.2) for 16 h at 30°C with shaking at 180 rpm. Then 40 μl of the culture was dropped on the LB agar in bottles (18 × 18 × 3 cm^3^) and cultured for 3 days at 30°C. The VOCs produced in the bottles were extracted and detected commercially by the solid-phase micro-extraction (SPME) method (Agilent, 6890N-5975B MSD, USA) (Gu et al., 2007). Briefly, SPME fibers were introduced into the atmosphere of the bottle to absorb the VOCs. Then, the SPME fibers with VOCs were incubated at 70°C for 20 min and were desorbed at 250°C for 5 min in the injection port using a DB-WAX column (30 m × 0.25 mm × 0.25 μm - polar, J&W Scientific, Folsom, CA). The initial oven temperature was 40°C (held for 2 min), ramped at 2°C/min to 220°C, and finally ramped at 20°C/min to 240°C (held for 10 min). GC–MS runs were performed for a total of 103 min. Compounds were identified by comparing their retention indices (RI) determined relatively to a homologous series of pure n-alkane standards or values reported in the literature (Jennings and Shibamoto, 1980). Fragmentation patterns in the mass spectra were also compared with those available in the Library of the National Institute of Standards and Technology (NIST05). The effective compounds were identified and estimated according to the methods described in the literature (Li et al., 2018; Chowdhary and Sharma, 2020). Experiments for treatments and controls were repeated three times per group.

### Statistical Analysis

The data obtained from the plant growth promotion test were statistically analyzed by ANOVA, and the mean values of the treated samples were separated by the least significant difference (LSD) test at *p* < 0.05 using SPSS version 19.0 (SPSS Inc., USA). Experiments were done in 10 replicates, and each experiment was repeated for three times. The results of repeated trials of each experiment were similar. Therefore, one representative trial for each experiment is reported in the Section Results.

## Results

### Effects of Strain TOR3209 Inoculation and VOCs on the Growth of Plant

**Figure 1:**
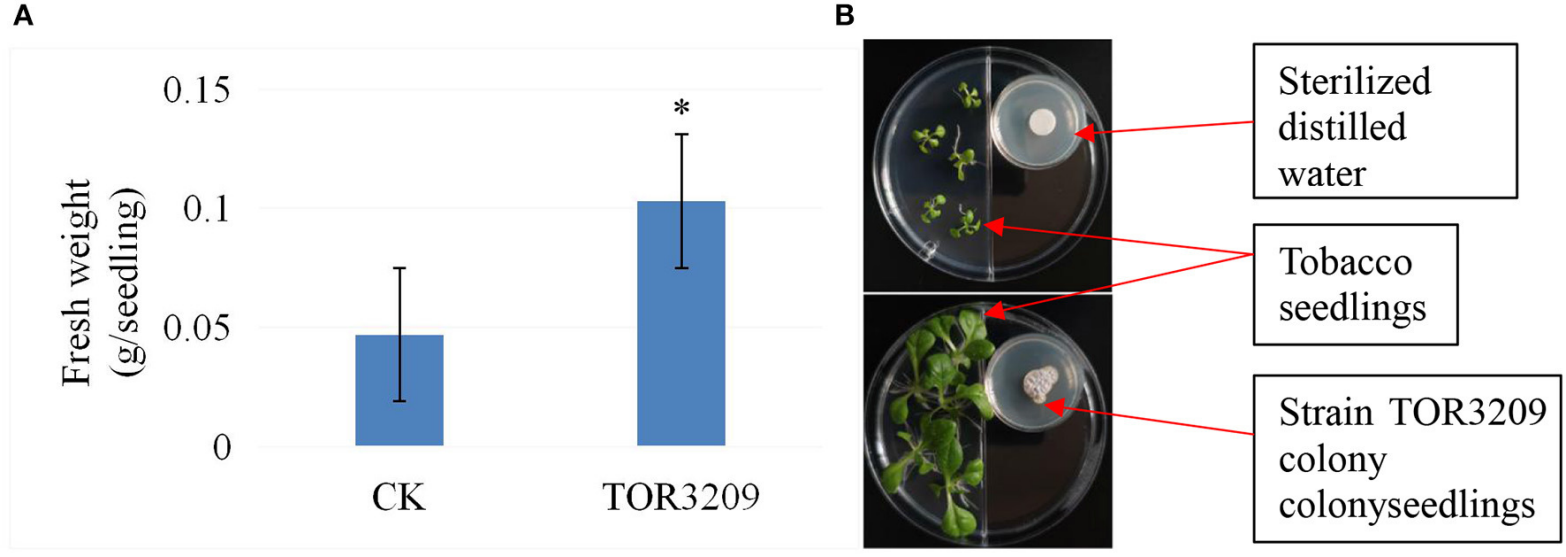
Comparison of effects of strain TOR3209 VOCs on tobacco growth. **(A)** Comparison of fresh weight between the CK seedlings and treatment seedlings; **(B)** Photographs showing the growth promotion on growth of tomato seedlings by strain TOR3209 co-culture (bellow) in comparison with CK control (upper). * The same letter followed the values in the same column means no significant difference based on the least significant difference (LSD) test (*p* < 0.05).

In the growth tests (Supplementary Table 1), a tendency toward the promotion of growth was observed in the tobacco seedlings. The seedlings grown in MS medium inoculated with strain TOR3209 showed a 20% increase in their total fresh weight after 5 days of inoculation, in comparison with the controls. However, the increase in growth was not significant according to the LSD test. In I-plates, the total fresh weight of tobacco seedlings co-incubated with strain TOR3209 was significantly elevated up to 100% (Figure 1).

### PGP Traits of *Streptomyces* sp. TOR3209

The analysis of IAA production showed that strain TOR3209 produced 12.06 μg/ml of IAA in the culture. In the P solubilization test, the strain TOR3209 grew slowly and weakly on the PKO medium. After 2–7 days of incubation, the colonies gradually expanded, and the phosphate solubilizing ring gradually appeared, but the ring was not clear and remained unchanged after 7 days. On organophosphorus medium, strain TOR3209 presented a more obvious dissolving effect. After 5 days of incubation, the solubilization ring was very obvious (SI = 1.6), and it continuously expanded until the entire plate became almost transparent. On CAS medium, colonies of strain TOR3209 began to grow after 1 week of incubation with the appearance of yellow circles, indicating the production of siderophores. Based on the result of the FeCl_3_ test, the siderophores produced by the strain TOR3209 were hydroxamate type. Detailed results for PGP traits of *Streptomyces* sp. TOR3209 are available in Supplementary Figure 1.

### Transcriptomal Analysis of Tobacco Plants Responding to VOCs

**Figure 2:**
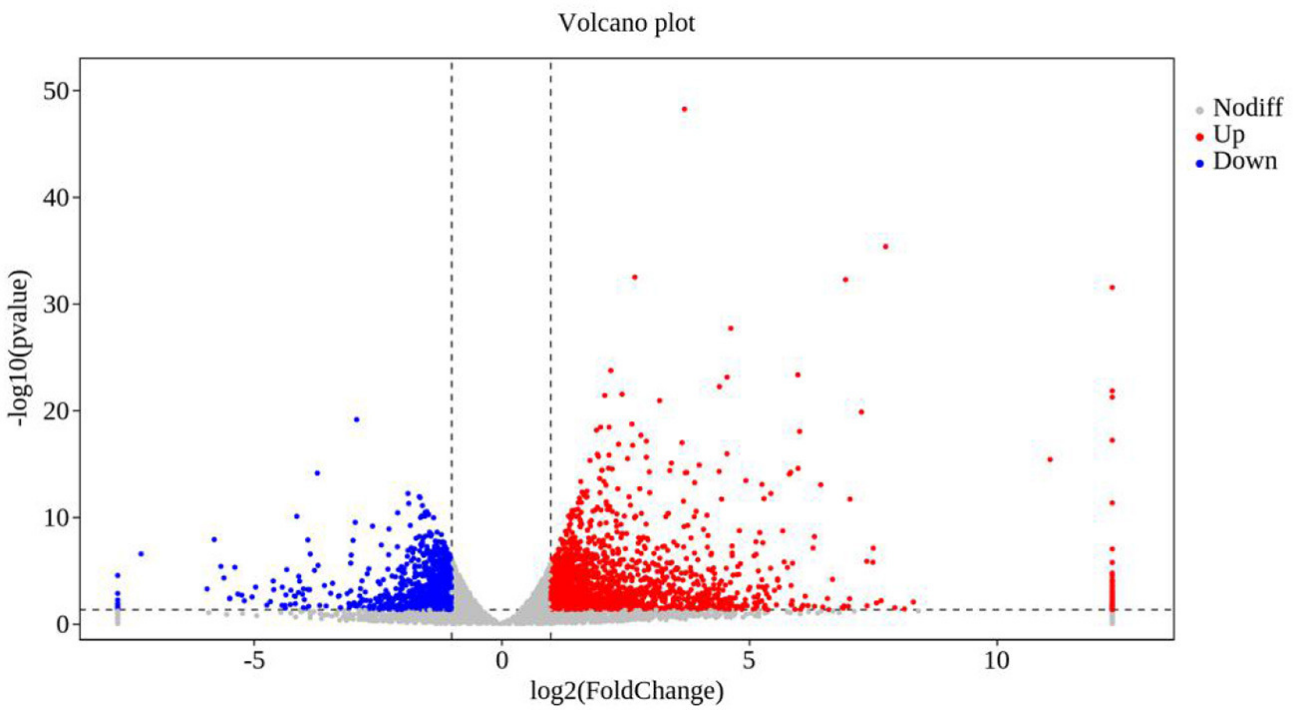
Volcanic diagrams of differential expression genes. The horizontal coordinates are log2 (fold change) and the ordinates are -log10 (*p*-value). The two vertical dashed lines are 2 times the difference threshold, and the horizontal dashed lines are *p*-value 0.05 thresholds. A red dot indicates the upregulated genes group, a blue dot indicates the downregulated genes group, and a gray dot represents a non-significant difference in the expression of the genes group. The scale was fixed considering the minimum and maximum log2 values (between −7.74 and 12.33) included in the Supplementary Tables 1, 2.

In transcriptomal analysis, 38,254 genes were detected in the plants co-cultured with strain TOR3209, and 37,636 genes were detected in the control plants. Analysis of RNAseq data revealed that 3,191 differentially expressed transcripts (DEGs) were induced by the VOCs of strain TOR3209, including 2,155 up-regulated and 1,036 down-regulated transcripts (Figure 2). The most important (Log2 fold change >5 or < -4) up-regulated (52) and down-regulated (23) transcripts are listed in Table 1. Among them, UDP-glycosyltransferase, glutamate receptor, RING/U-box protein with C6HC-type zinc finger, and leucine-rich repeat receptor-like protein kinase family protein were the highest up-regulated DEGs in the VOC-treated tobacco seedlings. Chaperone protein ClpB, MYB-like transcription factor family protein, and 17.3 kDa class II heat shock proteins were significantly repressed by the VOC treatment.

**Table 1:** Most important differentially expressed genes in tobacco plants inoculated with TOR3209.

**Gene ID**	**Log2 fold change**	**Gene description**
**Up-regulated genes**		
Niben101Scf00107g03008	12.33288866	Cysteine-rich venom protein
Niben101Scf02349g03003	11.07906488	Peroxidase 4
Niben101Scf00661g00002	8.319303503	UDP-glycosyltransferase 74 F1 LENGTH = 449
Niben101Scf08670g00020	8.139389486	Glutamate receptor 2.9
Niben101Scf07123g01015	7.759232431	Leucine-rich repeat receptor-like protein kinase family protein LENGTH = 1,045
Niben101Scf01463g08009	7.667222902	Calmodulin binding protein-like LENGTH = 494
Niben101Scf01942g04001	7.576358542	WRKY transcription factor 44
Niben101Scf02188g00002	7.509948729	WRKY transcription factor 1
Niben101Scf08526g02006	7.503570319	WRKY transcription factor 1
Niben101Scf12084g00003	7.382594655	WRKY transcription factor 1
Niben101Scf01400g00014	7.377702267	Thaumatin-like protein
Niben101Scf02115g04004	7.269910522	Eukaryotic aspartyl protease family protein LENGTH = 474
Niben101Scf12266g09001	7.039494994	Tetratricopeptide repeat (TPR)-like superfamily protein LENGTH=305
Niben101Scf02406g00002	7.031384344	GDSL esterase/lipase
Niben101Scf07589g01006	7.006395689	conserved hypothetical protein (*Ricinus communis*) gb|EEF44100.1| conserved hypothetical protein (*Ricinus communis*)
Niben101Scf06916g01011	6.949992145	NAC domain-containing protein 2
Niben101Scf13429g04019	6.919290018	L-lactate dehydrogenase A-like 6B
Niben101Scf05057g04016	6.905727119	WRKY transcription factor 1
Niben101Scf00892g00003	6.885022202	VQ motif protein (*Medicago truncatula*)
Niben101Scf06301g01003	6.687192872	Aspartic proteinase A1
Niben101Scf00096g00004	6.612642405	Myb family transcription factor APL
Niben101Scf04894g00006	6.58495066	UDP-glucose 4-epimerase
Niben101Scf08351g01002	6.490137969	Tumor susceptibility gene 101 protein
Niben101Scf02041g00002	6.447680321	Chitinase 8
Niben101Scf15327g00001	6.323748925	Calcium-binding protein CML39
Niben101Scf08921g02023	6.32181607	Cathepsin B-like cysteine proteinase 6
Niben101Scf11071g04005	6.293992449	Calmodulin binding protein-like LENGTH = 451
Niben101Scf00332g06012	6.191447348	Pyrophosphate-energized vacuolar membrane proton pump
Niben101Scf04944g05002	6.134053016	WRKY transcription factor 18
Niben101Scf02411g00015	6.088949822	Receptor kinase 3 LENGTH = 850
Niben101Scf02175g06008	6.068739476	Alkaline/neutral invertase LENGTH = 617
Niben101Scf11182g00003	6.021299925	Endonuclease 2
Niben101Scf00149g12021	5.991619157	Pyruvate decarboxylase 4
Niben101Scf04869g03002	5.988250289	Glucan endo-1,3-beta-glucosidase
Niben101Scf02264g06032	5.929532592	receptor kinase 2 LENGTH = 847
Niben101Scf07058g04002	5.880166829	P-loop containing nucleoside triphosphate hydrolases superfamily protein LENGTH = 500
Niben101Scf05099g01002	5.873047764	Mitochondrial import inner membrane translocase subunit Tim17/Tim22/Tim23 family protein LENGTH = 178
Niben101Scf06603g03002	5.867685439	WRKY transcription factor 55
Niben101Scf01444g00001	5.851494959	Inositol-1,4,5-trisphosphate 5-phosphatase 1
Niben101Scf02132g02012	5.848879214	2-aminoethanethiol dioxygenase-like protein (*Medicago truncatula*)
Niben101Scf01001g00003	5.843684467	Glucan endo-1,3-beta-glucosidase
Niben101Scf00526g00018	5.814028502	NAC domain-containing protein 86
Niben101Scf03374g06002	5.777012957	Non-specific lipid transfer protein-like 1
Niben101Scf00870g16006	5.709724282	ATP-dependent RNA helicase eIF4A
Niben101Scf02877g02005	5.681180326	Strictosidine synthase 1
Niben101Scf16244g02026	5.609478878	30S ribosomal protein S19
Niben101Scf03773g00009	5.57985346	Ethylene-responsive transcription factor 9
Niben101Scf10126g00007	5.515465694	Arogenate dehydrogenase LENGTH = 640
Niben101Scf12102g01006	5.441617826	VQ motif protein (*Medicago truncatula*)
Niben101Scf03595g10007	5.436938026	60S acidic ribosomal protein P1
Niben101Scf13920g00005	5.405652462	Acyl-CoA N-acyltransferases (NAT) superfamily protein (*Arabidopsis thaliana*) putative alanine acetyl transferase (*Arabidopsis thaliana*) gb|AAD15401.1| putative alanine acetyl transferase (*Arabidopsis thaliana*) gb|ABD60717.1| (*Arabidopsis thaliana*) gb|AEC08624.1| GCN5-related N-acetyltransferase-like protein (*Arabidopsis thaliana*)
Niben101Scf01094g03014	5.380529892	Ethylene-responsive transcription factor 1B
**Down-regulated genes**		
Niben101Scf00495g00003	−7.742771091	Chaperone protein ClpB
Niben101Scf04889g00020	−7.269929829	myb-like transcription factor family protein LENGTH = 233
Niben101Scf04040g08015	−5.793913087	17.3 kDa class II heat shock protein
Niben101Scf06977g00014	−5.659566414	Chaperone protein ClpB
Niben101Scf04040g09011	−5.597721532	17.3 kDa class II heat shock protein
Niben101Scf10384g00015	−5.479676566	17.6 kDa class I heat shock protein
Niben101Scf08675g00002	−5.47869793	BAG family molecular chaperone regulator 6
Niben101Scf03114g03011	−5.377817272	Chaperone protein HtpG
Niben101Scf00298g00001	−5.316649482	Ethylene-responsive transcription factor 1
Niben101Scf05378g03025	−5.236085551	ATP-dependent zinc metalloprotease FtsH
Niben101Scf02175g06018	−5.185219051	BAG family molecular chaperone regulator 6
Niben101Scf04040g09016	−5.028860367	17.3 kDa class II heat shock protein
Niben101Scf10306g00005	−4.958344447	17.3 kDa class II heat shock protein
Niben101Scf02552g00013	−4.659113231	Subtilisin-like serine protease 2 LENGTH = 764
Niben101Scf10384g00016	−4.602974453	17.6 kDa class I heat shock protein
Niben101Scf02124g01003	−4.593158829	Heat shock 70 kDa protein
Niben101Scf07790g00006	−4.426069226	COBRA-like protein 10
Niben101Scf04410g09016	−4.419070922	Chaperone protein ClpB
Niben101Scf02267g00013	−4.344794817	17.3 kDa class II heat shock protein
Niben101Scf01412g00015	−4.328983756	17.7 kDa class II heat shock protein
Niben101Scf02913g00028	−4.256421593	Alpha-galactosidase
Niben101Scf09552g01024	−4.24630503	Protein kinase superfamily protein with octicosapeptide/Phox/Bem1p domain LENGTH = 1,054
Niben101Scf00117g02017	−4.1759724	Octic, putative isoform 1 (*Theobroma cacao*)

**Figure 3:**
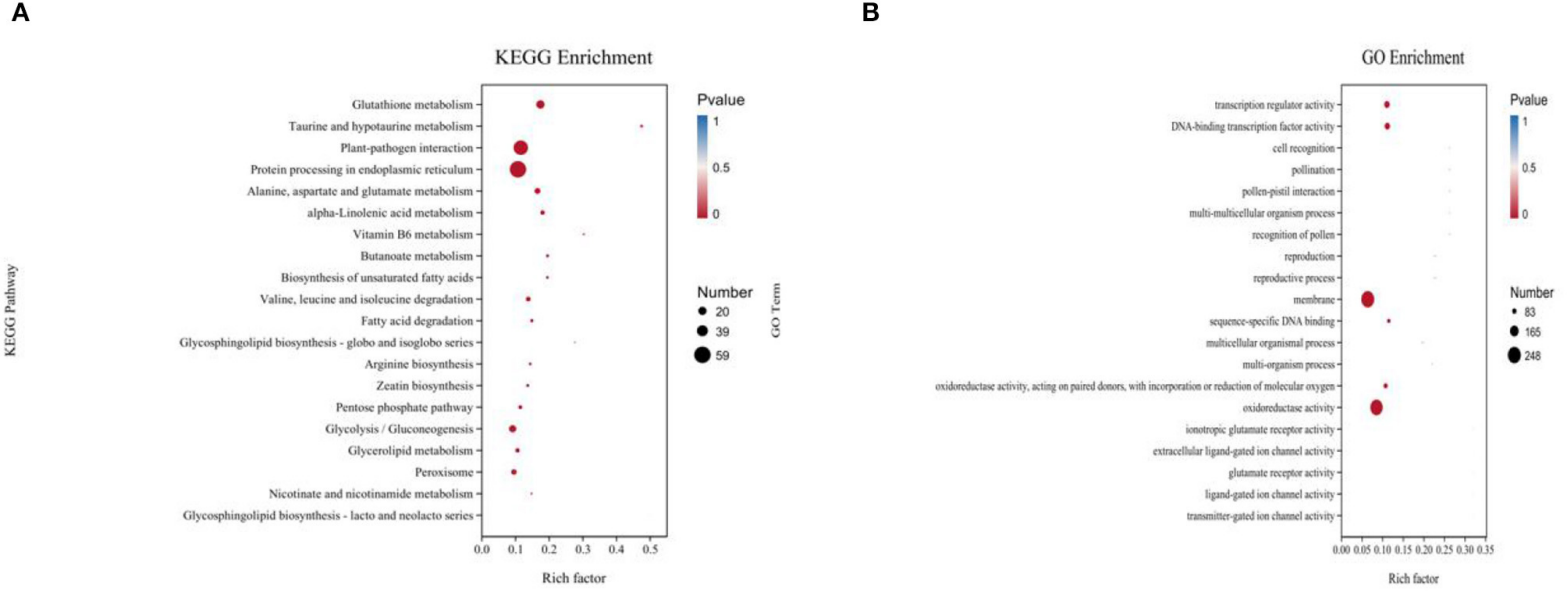
Gene ontology (GO) **(A)** and KEGG pathway-rich analysis **(B)**. Based on the GO-rich and KEGG results, the extent of richness is measured by the rich factor, false discovery rate (FDR) values, and the number of genes that were collected into this GO term or KEGG pathway. Rich factor refers to the ratio of the number of different genes collected in the GO term or KEGG pathway to the number of genes annotated.

**Figure 4:**
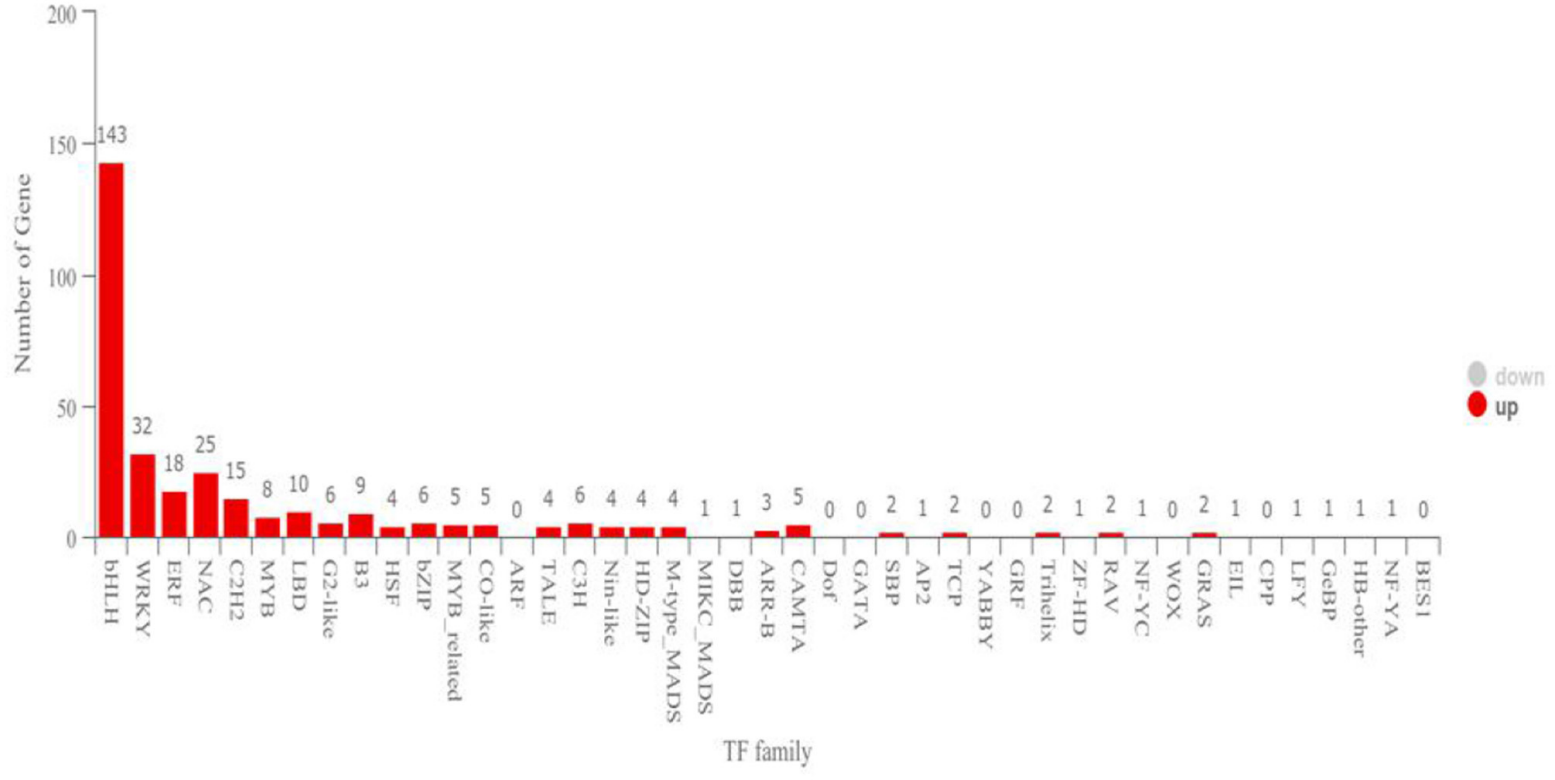
Transcription factor family. The horizontal coordinates are different transcription factor families, and the ordinates are the number of genes that fall into that transcription factor family.

In the functional annotation, DEGs with the largest increase in expression were those related to cell recognition, pollination, pollen–pistil interaction, multi-multicellular organism process, and recognition of pollen based upon the GO enrichment analyses (Figure 3; Table 1). Based on the KEGG pathway, the most significant up-regulated transcripts by VOCs were categorized as those related to glutathione metabolism, taurine and hypotaurine metabolism, and glycosphingolipid biosynthesis pathways (Figure 3). The expression profile of TF genes displays the upcoming transcription actions regulated by these genes. The largest TF families detected in our study were basic helix-loop-helix (bHLH) DNA-binding superfamily protein, WRKY, NAC domain-containing protein (NAC), ethylene-responsive factor (ERF), and zinc finger protein genes (C2H2) (Figure 4).

### Analysis of VOCs Produced by *Streptomyces* sp. TOR3209

**Figure 5:**
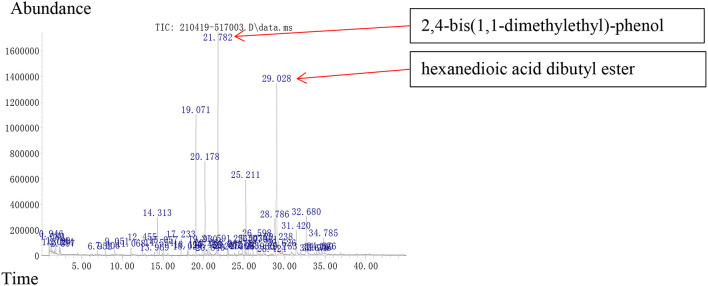
Volatile organic compounds of strain TOR3209 using GC-MS. The samples were incubated in bottle containing LB agar for 3 days. 2,4-bis(1,1-dimethylethyl)-phenol and hexanedioic acid dibutyl ester were detected.

In GC–MS analysis, 48 compounds were detected, among which eight (accounting for 15.22% of the total VOCs) were reported as PGP effective VOCs, and 14 (accounting for 37.36% of the total VOCs) had the potential to increase the resistance of the plant to phytopathogens (Supplementary Table 2). According to their relative abundances, seven compounds could be identified as the main components, each contributing to more than 5% of the total: the pathogen-inhibiting compounds like trans-1,10-dimethyl-trans-9-decalol (9.43%), 1,4,5,6,7,8,9,9a-octahydro-1,1,7-trimethyl-[3aR-(3a.alpha.,7.alpha.,9a.beta.)]- 3a,7-methano-3aH-cyclopentacyclooctene (6.29%), and 2,4-bis(1,1-dimethylethyl)- phenol (13.61%); the growth-promoting compounds like n-hexadecanoic acid (6.53%) (Chowdhary and Sharma, 2020); and the compounds of an unknown function like (-)-.alpha.-Panasinsen (5.12%), pentadecanoic acid (5.58%), and hexanedioic acid dibutyl ester (25.58%). Among these compounds, 2,4-di-tert-butylphenol [o 2,4-bis(1,1-dimethylethyl)- phenol] and dibutyl adipate (o hexanedioic acid dibutyl ester) were the most abundant VOCs produced by the strain TOR3209 (Figure 5).

## Discussion

It is well known that some microorganisms have the potential to promote plant growth through direct and/or indirect effects. The direct effects include the production of molecules that promote plant growth (Goswami et al., 2016). In this study, the growth (total fresh weight) of tobacco seedlings was significantly increased (100%) by co-culturing with the strain *Streptomyces* sp. TOR3209 (Figure 1; Supplementary Table 1), demonstrating that this strain produced VOCs as PGP agents, consistent with the previous reports for other microbial VOCs (Kanchiswamy et al., 2015). These results suggest an alternative mechanism for the PGP effects of strain TOR3209, other than the previously described regulation of the rhizosphere microbial community (Hu et al., 2020).

As reported previously, the VOCs mainly react with the aerial parts of the plants (Asari et al., 2016). Considering the low amount of VOCs, it could be hypothesized that they cannot function as nutrients, but can serve as signals to regulate the production of plant genes related to growth. Indeed, the transcriptome analysis based upon the KEGG analysis (Kanehisa and Goto, 2000) in the present study revealed that VOCs of strain TOR3209 up-regulated the expression of multiple genes to improve plant growth. The highest increase in the expression was noted for the genes of UDP-glycosyltransferase, glutamate receptor, RING/U-box protein with C6HC-type zinc finger, and leucine-rich repeat receptor-like protein kinase family protein in the VOC treatment samples, which indicated the possible relationships between these genes and the increased growth. The expression of UDP-glucuronosyltransferase (UGT) in meristematic cells is correlated with mitosis and is required for the normal development of plants (Woo et al., 1999). So, the VOCs of strain TOR3209 might increase plant growth by promoting cell division. Also, glycosylation is a universally important mechanism in certain organisms to control the impact of a wide range of hydrophobic compounds that can directly derange cellular metabolism (Nebert, 1991). Therefore, VOCs of strain TOR3209 might also promote plant growth by regulating the UGT-mediated glycosylation in plants. Plant glutamate receptor-like genes (GLRs) coded amino acid receptors related to plant defense and innate immune response (Forde and Roberts, 2014). Recently, the role of GLRs in controlling the plant height has also been established (Kaur, 2020), which further explained the PGP effects of VOCs produced by strain TOR3209. U-box proteins play important myriad functions in plants, such as in growth and development, cell cycle regulation, morphogenesis, stress resistance, and regulating the innate immune response of plants to biotic stress factors. Therefore, strain TOR3209 was capable of promoting growth through VOCs that induce the expression of genes coding U-box proteins and LRR-RLKs (Liu et al., 2017), which further regulate the expression of other genes related to the plant growth and development, including the synthesis of proteins and enzymes.

Transcription factors (TFs) play essential roles in plants, as well as in all other living organisms, by controlling the expression of genes involved in various cellular processes (Han et al., 2014). Numerous processes, such as brassinosteroid signaling, ABA signaling, and axillary meristem formation, are regulated by the members of the bHLH family of TFs during the seedling development (Zhao et al., 2012). A total of 143 transcripts were identified as bHLH TF proteins in the present study, which have been identified as phytochrome-interacting factors (Toledo-Ortiz et al., 2003) and constitute the largest TF family proteins that are produced during the interaction of tobacco plants with the VOCs of strain TOR3209. These results further indicated that the VOCs produced by strain TOR3209 were capable of promoting plant growth by regulating a serial synthesis of TFs. Another large NAC TF family was involved in inducing diverse developmental events in *Arabidopsis* and soybean (Shamimuzzaman and Vodkin, 2013). This NAC TF family has the capacity to permit crosstalk between different pathways (Olsen et al., 2005). In our present study, 25 putative NAC genes were detected during plant growth. Furthermore, genes encoding C_2_H_2_ zinc finger protein play a significant role in the development and differentiation (Razin et al., 2012). During the interaction, 15 putative C_2_H_2_ zinc finger genes were identified. In addition, ethylene-responsive factor (ERF) TFs belonging to the Apetala2/ERF TF family were also found to be involved in plant growth. The WRKY genes coding TFs were involved in a broad range of biological processes, including diverse biotic/abiotic stress responses, and developmental and physiological processes (Birkenbihl et al., 2017). A total of 18 putative ERF and 32 putative WRKY genes were detected during plant development in the present study. So, it is clear that the VOCs of *Streptomyces* sp. TOR3209 could regulate diverse metabolism pathways, which further improved the growth of tobacco plants.

The significantly down-regulated genes in the samples subjected to VOC treatment were chaperone protein ClpB, MYB-like transcription factor family protein, and 17.3 kDa class II heat shock proteins. These three genes and most of the other down-regulated genes are genes that confer adaptation to environmental stress. ClpB (caseinlytic proteinase) is related to the disaggregation of protein complexes and cell adaptation to heat stress (HS) (Mishra and Grover, 2016). A MYB-like transcription factor is involved in plant abiotic stress (like salt) tolerance and phytohormone signal transduction (Zhang et al., 2018). Furthermore, the 17.3 kDa class II heat shock proteins are also stress tolerance proteins related to heat shock (Kang et al., 2021). The reason why the expression of these genes was elevated by VOC treatment is not very clear, but it is possible that the VOCs shuttled down the expression of genes related to some unnecessary cell functions, while stimulating the expression of other genes essential for plant growth.

Previously, more than 120 VOCs have been detected in *Streptomyces*, which have been classified into different chemical groups, such as alkanes, alkenes, esters, alcohols, and ketones (Schöller et al., 2002). According to the literature, 15.22% (eight) of the VOC molecules detected in the present study presented PGP effects, which might be the potential compounds directly responsible for the growth promotion of tobacco seedlings in the I-plates co-cultured with strain TOR3209. Meanwhile, more VOCs (37.36%, 14 molecules) were related to the resistance to phytopathogens; however, it is not clear whether these VOCs can also promote the growth of plants. As the most abundant compound (25.58%) in the VOCs of strain TOR3209, the effects of hexanedioic acid dibutyl ester on the plant has not been reported and its function in regulating plant growth deserves further study.

In the PGP trait analysis, 12.06 μg/ml of IAA production by strain TOR3209 was recorded, which is relatively a high level since values around 5 to 10 μg/ml are commonly reported. However, some actinobacterial strains capable of producing more than 40 μg/ml have been isolated (Vasconcellos et al., 2010). In addition, the IAA production efficiency of strain TOR3209 might be increased by optimizing the culture conditions, because the production of IAA by a *Streptomyces* strain was increased from 20.46 to 82.36 μg/ml by modifying the medium components (Myo et al., 2019). Therefore, strain TOR3209 could be a good stimulator of plant growth via the production of IAA, as described for other *Streptomyces* strains and other bacteria (Hayat et al., 2010). Another PGP trait of strain TOR3209 is to dissolve phosphate, particularly the organic phosphate, which could subsequently promote plant growth (Spaepen et al., 2007). In this study, we also proved the ability of strain TOR3209 to produce siderophore, a low-molecular-weight iron-binding compound that could help the plant chelate iron in the Fe^3+^ restricted soil (Bottini et al., 2004) and inhibit plant pathogens due to their competitive role (Otieno et al., 2015). Currently, many siderophore molecules, with diverse chemical structures and different bioactivities against a wide spectrum of pathogenic bacteria, have been isolated from *Streptomyces* species (Terra et al., 2021). Different from the previous reports that the *Strteptomyces tendae* Tü 901/8c and *Streptomyces* sp. Tü 6125 isolated from soils produced the catecholate siderophore (Fiedler et al., 2001), the strain TOR3209 produced the hydroxamate siderophore, indicating that different *Streptomyces* species might have distinct pathways for the synthesis of siderophore. Although the PGP features of strain TOR3209 were detected in the present study, they might not contribute to the growth of tobacco seedlings in the inoculation test in the present study, since they are not necessary for the plants grown in the sterilized medium, which is different from the situation in soils (Hu et al., 2020). However, the capacity of *Streptomyces* species to produce IAA and siderophore deserves further study to explore their biofertilizer and biopesticide properties.

In our present study, the promotion of plant growth was not statistically significant following the direct inoculation of strain TOR3209, but it was significant in VOC treatments (Supplementary Table 1). This was inconsistent with the previous results that inoculation with strain TOR3209 significantly promoted the growth of tomato plants (Hu et al., 2020). This discrepancy might be related to the differences in the culture conditions because the inoculated plants were cultured in soil (Hu et al., 2020), while the experiments in our study were carried out in medium or artificial substrate. In the medium, the rhizosphere microbiome is absent and the nutrients are not limited, which might also explain why the direct inoculation with the strain could not significantly improve the growth of tobacco seedlings in the present study. Furthermore, plant genotypes (Schwachtje et al., 2012) and environmental conditions (Martínez et al., 2011) also affect the inoculation effects of PGPR, and even the PGP effects of microbial siderophore could be different for distinct tobacco varieties (Robin et al., 2006).

## Conclusion

The VOCs of strain TOR3209 could improve the growth of the tested plants and induce the up-regulated expression of a key group of genes associated with plant growth and development and the down-regulation of some adaptation genes. Remarkably, our results suggest that the genes related to UDP-glycosyltransferase, glutamate receptor, RING/U-box protein with C6HC-type zinc finger, and leucine-rich repeat receptor-like protein kinase family protein presented greatly up-regulated expression in the tobacco seedlings in response to the VOCs of strain TOR3209, and all these genes were involved in the plant growth and development processes. In addition, the important VOCs of strain TOR3209 were found to be 2,4-bis(1,1-dimethylethyl)-phenol and hexanedioic acid dibutyl ester (with two compounds exhibiting antifungal functions), and their other PGP effects need to be further investigated. In the long term, these new findings would facilitate the development of biofertilizers and biopesticides.

## Data Availability Statement

The datasets presented in this study can be found in online repositories. The names of the repository/repositories and accession number(s) can be found at: National Center for Biotechnology Information (NCBI) BioProject database under accession number PRJNA814410.

## Author Contributions

YH conceived the study and wrote the manuscript. YH and ZW designed the experiments. YH, WG, JP, JG, JM, XW, CZ, NJ, EW, DH, and ZW did the experiments. YH and WG assisted in the data analysis. YH, EW, and JG edited the article. All authors contributed to the article and approved the submitted version.

## Funding

This work was supported by the National Key Research and Development Program of China (2021YFD1901004-4), the HAAFS Science and Technology Innovation Special Project (2022KJCXZX-ZHS-3), and the Project of Natural Science Foundation of Hebei Province (C2020301047). EW was supported by the Project of Sabbatical Year and SIP20200726 authorized by IPN, Mexico.

## Conflict of Interest

The authors declare that the research was conducted in the absence of any commercial or financial relationships that could be construed as a potential conflict of interest.

## Publisher's Note

All claims expressed in this article are solely those of the authors and do not necessarily represent those of their affiliated organizations, or those of the publisher, the editors and the reviewers. Any product that may be evaluated in this article, or claim that may be made by its manufacturer, is not guaranteed or endorsed by the publisher.
