# Supplementary material for: Volatile Organic Compounds of Streptomyces sp. TOR3209 Stimulated Tobacco Growth by Up-Regulating the Expression of Genes Related to Plant Growth and Development
Source: Front Microbiol. 2022 May 20;13:891245. doi: 10.3389/fmicb.2022.891245 (PMC9164152; doi:10.3389/fmicb.2022.891245)
Supplement: Supplementary file 1 [file Table_1.pdf]

Supplementary tables

Supplementary Table 1. Growth promoting of tobacco by TOR3209 inoculation*

Treatment	Total fresh weight (g)	
	Inoculation	Co-culture
CK	0.10a	0.05a
TOR3209	0.12a	0.10b
Increase	20%	100%

*. The same letter followed the values in the same column means no significant difference based on the least significant difference (LSD) test ($p < 0.05$).

Supplementary Table 2. VOCs detected from the metabolites of *Streptomyces* sp. TOR3209

R.T. (min)	VOCs	Effect	Description of effects	Relative abundance	Reference
0.946	Carbon dioxide	Disease resistance	Production of volatile metabolites from *Streptomyces albidoflavus* cultivated on gypsum board and tryptone glucose extract agar-influence of temperature, oxygen and carbon dioxide levels.	1.84%	(Sunesson et al., 1997)
1.08	Ethyl alcohol	Disease resistance	Identification of rhizospheric actinomycete *Streptomyces lavendulae* SPS-33 and the inhibitory effect of its volatile organic compounds against *Ceratocystis fimbriata* in postharvest sweet potato (*Ipomoea batatas*)	1.35%	(Li et al., 2020)
1.302	Acetic acid	Unknown		0.53%	
1.799	Silanediol, dimethyl-	Unknown		0.28%	
2.244	1-Butanol, 3-methyl-	PGPR	Effects of 3-methyl-1-butanol on seed germination and seedling growth of maize and wheat	0.51%	(Li et al., 2018)
2.397	Disulfide, dimethyl	Disease resistance	Fumigant activity of volatiles of Streptomyces globisporus JK-1 against Penicillium italicum on Citrus microcarpa	0.42%	(Li et al., 2010)
6.952	Dimethyl trisulfide	Disease resistance	Identification of Rhizospheric Actinomycete Streptomyces lavendulae SPS-33 and the Inhibitory Effect of its Volatile Organic Compounds against Ceratocystis fimbriata in Postharvest Sweet Potato (Ipomoea batatas）	0.33%	(Li et al., 2020)
9.051	Benzeneacetaldehyde	Unknown		0.77%	
11.068	Phenylethyl Alcohol	PGPR	Identification of microbial volatile organic compounds and their effect in A. thaliana's growth	0.74%	(Rivas et al., 2018)

12.455	Cyclopentasiloxane, decamethyl-	Disease resistance	Identification of Rhizospheric Actinomycete Streptomyces lavendulae SPS-33 and the Inhibitory Effect of its Volatile Organic Compounds against Ceratocystis fimbriata in Postharvest Sweet Potato (Ipomoea batatas）	0.53%	(Li et al., 2020)
13.969	2-Cyclopenten-1-one, 3,4-dimethyl-	Disease resistance	Geosmin and Related Volatiles in Bioreactor-Cultured Streptomyces citreus CBS 109.60）	0.25%	(Pollak and Berger, 1996)
14.313	1H-Indene, 1-ethylideneoctahydro-7a-methyl-, cis-	Unknown		2.44%	
14.554	Cyclohexane, 1,1,4,4-tetramethyl-2,5-dimethylene-	Disease resistance	Effect of volatile substances of Streptomyces platensis F-1 on controlof plant fungal diseases	0.58%	(Wan et al., 2008)
15.057	cis,cis,cis-Perhydroacenaphtene	Unknown		0.68%	
18.028	5-Eicosene, (E)-	Unknown		0.31%	
18.117	n-Decanoic acid	Unknown		0.41%	
19.071	trans-1,10-Dimethyl-trans-9-decalol	Disease resistance	Effect of volatile substances of Streptomyces platensis F-1 on controlof plant fungal diseases	9.43%	(Wan et al., 2008)
19.93	Benzene, 1-(1,1- dimethylethyl)-4-methoxy-	Disease resistance	Effect of volatile substances of Streptomyces platensis F-1 on controlof plant fungal diseases	0.81%	(Wan et al., 2008)
20.178	3a,7-Methano-3aH-cyclopentacyclooctene, 1,4,5,6,7,8,9,9a- octahydro-1,1,7-trimethyl-, [3aR-(3a.alpha.,7.alpha.,9a.beta.)]-	Disease resistance	Effect of volatile substances of Streptomyces platensis F-1 on controlof plant fungal diseases	6.29%	(Wan et al., 2008)
20.465	Azulene, 1,2,3,5,6,7,8,8a – octahydro-1,4-dimethyl-7-(1-methylethenyl)-, [1S-(1.alpha.,7.alpha.,8a.beta.)]-	Disease resistance	Effect of volatile substances of Streptomyces platensis F-1 on controlof plant fungal diseases	0.61%	(Wan et al., 2008)
20.668	Propanamide, N-(4-methoxyphenyl)-2,2-dimethyl-	Unknown		0.55%	
20.84	1-Dodecanol	Unknown		0.40%	
21.782	Phenol, 2,4-bis(1,1-dimethylethyl)-	Disease resistance	Effect of volatile substances of Streptomyces platensis F-1 on controlof plant fungal diseases	13.61%	(Wan et al., 2008)
22.952	Dodecanoic acid	Unknown		0.78%	
23.079	2-Tetradecanone	Unknown		0.47%	
24.358	2-Naphthalenemethanol, 1,2,3,4,4a,5,6,7-octahydro-.alpha.,.alpha.,4a,8-tetramethyl-,	PGPR	Study of plant growth regulating activity of 2-[(1-naphthalen-1-yl-ethylimino)-methyl]-phenol and its transition metal complexes on trigonella foenum-graecum.	0.33%	(Palande and Swamy, 2017)

	(2R-cis)-				
24.778	2-Naphthalenemethanol, 1,2,3,4,4a,5,6,8a-octahydro-.alpha.,.alpha.,4a,8-tetramethyl-, [2R-(2.alpha.,4a.alpha.,8a.beta.)]-	Unknown		0.45%	
25.083	Bicyclo[4.4.0]dec-1-ene, 2-isopropyl-5-methyl-9-methylene-			0.37%	
25.211	(-)-.alpha.-Panasinsen	Unknown		5.12%	
26.07	Heptadecane	Disease resistance	Identification of Rhizospheric Actinomycete Streptomyces lavendulae SPS-33 and the Inhibitory Effect of its Volatile Organic Compounds against Ceratocystis fimbriata in Postharvest Sweet Potato (Ipomoea batatas）	1.05%	(Li et al., 2020)
26.598	Tetradecanoic acid	PGPR	Effects of Tetradecanoic Acid on Growth and Activities of Leaf Defense Enzymes of Eggplant Seedlings	1.51%	(Zhou et al., 2011)
26.96	Phenol, 4-amino-3-methyl-	Unknown		0.26%	
27.361	Tetradecanoic acid	Unknown		1.51%	
28.424	Pyrrolo[1,2-a]pyrazine-1,4-dione, hexahydro-3-(2-methylpropyl)-	Disease resistance	Volatile organic compounds produced by the phytopathogenic bacterium Xanthomonas campestris pv. vesicatoria 85-10	0.26%	(Weise et al., 2012)
28.786	Pentadecanoic acid	Unknown		5.54%	
29.028	Hexanedioic acid, dibutyl ester	Unknown		25.58%	
29.238	2-Pentadecanone, 6,10,14-trimethyl-	PGPR	Seedling growth-promoting composition, a kit, a method for applying the former, and use of the volatile organic compounds comprising the composition	2.05%	(Cortez et al.)
29.626	Pentadecanoic acid	Unknown		5.54%	
29.753	Ethyl tridecanoate	Unknown		0.47%	
31.42	Tridecaldehyde	PGPR	Plant growth-promoting rhizobacteria modulate root-system architecture in Arabidopsis thaliana through volatile organic compound emission	3.52%	(Gutiérrez-Luna et al., 2010)
32.68	n-Hexadecanoic acid	PGPR	Plant Growth Promotion and Biocontrol Potential of Fungal Endophytes in the Inflorescence of Aloe vera L.	6.53%	(Chowdhary and Sharma, 2020)
33.946	Pentafluoropropionic acid, hexadecyl ester	Unknown		0.38%	
34.219	2-Cyclohexen-1-ol	PGPR	Pyrazole plant growth regulants	0.39%	(Johnson and Sweetser, 1976)
34.576	Heptadecanoic acid	Unknown		1.76%	

Chowdhary, K., and Sharma, S. (2020). Plant Growth Promotion and Biocontrol Potential of Fungal Endophytes in the Inflorescence of Aloe vera L. *Proceedings of the National Academy of Sciences, India Section B: Biological Sciences* 90(5), 1045-1055.

Cortez, A.Q., Robles, P.F., and Ibañez, M.P. "SEEDLING GROWTH-PROMOTING COMPOSITION, A KIT, A METHOD FOR APPLYING THE FORMER, AND USE OF THE VOLATILE ORGANIC COMPOUNDS COMPRISING THE COMPOSITION".).

Gutiérrez-Luna, F., López-Bucio, J., Altamirano-Hernández, J., Valencia-Cantero, E., Cruz, H., and Macías-Rodríguez, L. (2010). Plant growth-promoting rhizobacteria modulate root-system architecture in Arabidopsis thaliana through volatile organic compound emission. *Symbiosis* 51(1), 75-83.

Johnson, A.L., and Sweetser, P.B. (1976). "Pyrazole plant growth regulants".).

Li, C., Zhang, J., Zhao, C., Jiang, H., Ren, X., Su, W., et al. (2018). Effects of 3-methyl-1-butanol on seed germination and seedling growth of maize and wheat. *Bulletin of Botanical Research* 38(5), 785-789.

Li, Q., Ning, P., Zheng, L., Huang, J., Li, G., and Hsiang, T. (2010). Fumigant activity of volatiles of Streptomyces globisporus JK-1 against Penicillium italicum on Citrus microcarpa. *Postharvest Biology and Technology* 58(2), 157-165. doi: https://doi.org/10.1016/j.postharvbio.2010.06.003.

Li, X., Li, B., Cai, S., Zhang, Y., Xu, M., Zhang, C., et al. (2020). Identification of Rhizospheric Actinomycete Streptomyces lavendulae SPS-33 and the Inhibitory Effect of its Volatile Organic Compounds against Ceratocystis fimbriata in Postharvest Sweet Potato (Ipomoea batatas (L.) Lam.). *Microorganisms* 8(3). doi: 10.3390/microorganisms8030319.

Palande, S.V., and Swamy, D. (2017). STUDY OF PLANT GROWTH REGULATING ACTIVITY OF 2-[(1-NAPHTHALEN-1-yl-ETHYLIMINO)-METHYL]-PHENOL AND ITS TRANSITION METAL COMPLEXES ON TRIGONELLA FOENUM-GRAECUM. TRITICUM AESTIVUM AND BRASSICA NIGRA. *Acta Ciencia Indica* 43(3), 425-430.

Pollak, F.C., and Berger, R.G. (1996). Geosmin and Related Volatiles in Bioreactor-Cultured Streptomyces citreus CBS 109.60. *Applied and Environmental Microbiology* 62(4), 1295-1299. doi: doi:10.1128/aem.62.4.1295-1299.1996.

Rivas, G.L., Camarena-Pozos, D.A., Pérez, M., Cabriales, J., and Martinez, L. (2018). Identification of microbial volatile organic compounds and their effect in A. thaliana's growth. *Ingeniería e Investigación* 3(30), 41.

Sunesson, A.-L., Nilsson, C.-A., Carlson, R., Blomquist, G., and Andersson, B. (1997). Production of volatile metabolites from streptomyces albidoflavus cultivated on gypsum board and tryptone glucose extract agar-influence of temperature, oxygen and carbon dioxide levels. *The Annals of Occupational Hygiene* 41(4), 393-413. doi: https://doi.org/10.1016/S0003-4878(96)00046-4.

Wan, M., Li, G., Zhang, J., Jiang, D., and Huang, H.C. (2008). Effect of volatile substances of Streptomyces platensis F-1 on control of plant fungal diseases. *Biological Control* 46(3), 552-559.

Weise, T., Kai, M., Gummesson, A., Troeger, A., von Reuß, S., Piepenborn, S., et al. (2012). Volatile organic compounds produced by the phytopathogenic bacterium Xanthomonas campestris pv. vesicatoria 85-10. *Beilstein Journal of Organic Chemistry* 8(1), 579-596.

Zhou, B., Jianxia, W.U., Yuling, Y., Lin, H., and Chuanqi, S. (2011). Effects of Tetradecanoic Acid on Growth and Activities of Leaf Defense Enzymes of Eggplant Seedlings. *Acta Agriculturae Boreali-Occidentalis Sinica*.
