# Supplementary material for: Volatile Organic Compounds of Streptomyces sp. TOR3209 Stimulated Tobacco Growth by Up-Regulating the Expression of Genes Related to Plant Growth and Development
Source: Front Microbiol. 2022 May 20;13:891245. doi: 10.3389/fmicb.2022.891245 (PMC9164152; doi:10.3389/fmicb.2022.891245)
Supplement: Supplementary file 2 [file Image_1.pdf]

Supplementary Figure

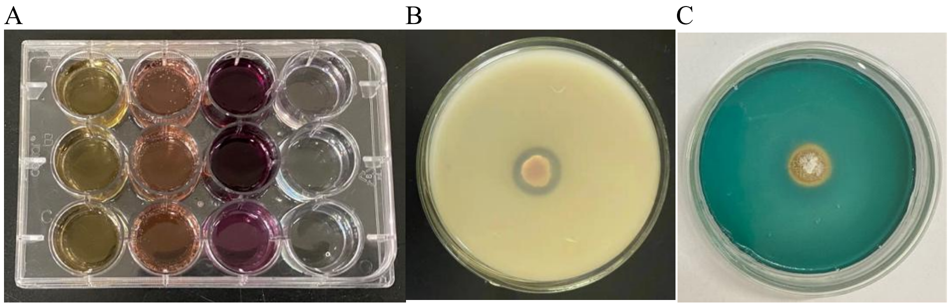

Fig. 1. Tests for the PGPR traits of *Streptomyces* sp. TOR3209. A: Auxin production; B: Dissolving organophosphate function; C: Siderophore production.
